# DCTable: A Dilated CNN with Optimizing Anchors for Accurate Table Detection

**DOI:** 10.3390/jimaging9030062

**Published:** 2023-03-07

**Authors:** Takwa Kazdar, Wided Souidene Mseddi, Moulay A. Akhloufi, Ala Agrebi, Marwa Jmal, Rabah Attia

**Affiliations:** 1 Sercom Laboratory, Ecole Polytechnique de Tunisie, Université de Carthage, La Marsa 2078, Tunisia; 2 Perception, Robotics, and Intelligent Machines (PRIME), Department of Computer Science, Université de Moncton, Moncton, NB E1A 3E9, Canada

**Keywords:** dilated convolutions, anchors, bilinear interpolation, table detection, Faster R-CNN

## Abstract

With the widespread use of deep learning in leading systems, it has become the mainstream in the table detection field. Some tables are difficult to detect because of the likely figure layout or the small size. As a solution to the underlined problem, we propose a novel method, called DCTable, to improve Faster R-CNN for table detection. DCTable came up to extract more discriminative features using a backbone with dilated convolutions in order to improve the quality of region proposals. Another main contribution of this paper is the anchors optimization using the Intersection over Union (*IoU*)-balanced loss to train the RPN and reduce the false positive rate. This is followed by a RoI Align layer, instead of the ROI pooling, to improve the accuracy during mapping table proposal candidates by eliminating the coarse misalignment and introducing the bilinear interpolation in mapping region proposal candidates. Training and testing on a public dataset showed the effectiveness of the algorithm and a considerable improvement of the *F*1-score on ICDAR 2017-Pod, ICDAR-2019, Marmot and RVL CDIP datasets.

## 1. Introduction

The wide use of paper documents in several domains such as finance, business and sciences has pushed researchers to develop digitization solutions and invest in its related technologies, from scanning to data extraction. In this context, Document Image Analysis and Recognition (DIAR) systems were designed to reduce human efforts and errors in information extraction from scanned documents [1]. Therefore, various processes, including invoice processing in manufacturing, have become automatic. Actually, in an invoice with a sophisticated template, the data are not narrative but organized in tables. Therefore, there is a need for the accurate extraction of data presented in tables. Table detection was always considered as a part of the document image analysis process [2] performed in a prepossessing step for OCR (Optical Character Recognition). For this purpose, a great deal of table detection techniques were proposed for several formats of documents (PDF or raster images [3]). While PDF is a vectorized representation that facilitates document reproduction to devices, such as a printer, the raster image is produced by a scanner or camera-capture and represented by pixels [4]. The table detection is a well-studied topic in the area of the document analysis community. Regardless of its layout, it is quite easy for humans to find and read a table in a document. However, for an algorithm, it is more difficult for two reasons. The first one is the high intra-class variance of tables where the system has to cope with different layouts and sizes as it could be missing ruling lines, nested rows and columns, etc., especially when it comes to small tabular regions. The second reason is the low inter-class variance between tables where other data containers, such as figures and charts, risk being mistakenly localized/classified as tables due to the similarity among them. This risk could affect successful data extraction workflow and yet increases false positives.

Driven by the enthusiasm on region-based CNN (R-CNN) [5] and its further improvements, many researchers took advantage of the novel blend of R-CNN in various tasks. Fast R-CNN [6] generates region proposals from extracted feature maps and reshapes them into a fixed size using a ROI pooling layer. Fast R-CNN is comparatively fast to R-CNN in both training and tests. Faster R-CNN [7] came up with an object detection algorithm that eliminates the selective search algorithm and allows the region proposal network (RPN) to learn the region proposals. A RPN is a fully convolutional network that predicts positions and probability scores for each region proposal [7]. RPN and Fast R-CNN are merged into a single network so that the RPN component tells the Fast R-CNN where to look. By introducing the valuable region proposal networks (RPN) [6], Faster R-CNN [7] gained a large amount of interest from the table detection community [3]. In 2017, the very first deep learning table detection approach was proposed in [8], where authors used the Faster R-CNN [7]. Faster R-CNN was extended by the Mask R-CNN [9] with a branch for predicting an object mask in parallel with the segmentation masks on each RoI (Region of Interest) for bounding box recognition. Then, many works adopted the Mask R-CNN [9] for more accurate table detection tasks. Since then, researchers in the table detection community have started to use a number of efficient developed deep learning frameworks, such as in [10,11].

Despite the impressive results of Faster R-CNN in table detection, this task still remains a serious challenge. The confusion problem between tables and charts produces a considerable number of false positives and consequently, affects the performance. Moreover, small tabular regions also represent a serious problem in the table detection since they risk being classified as background. When analyzing the Faster R-CNN, we noticed the following problems: (i) It is true that the region proposal network (RPN) is designed to generate region proposals with different scales based on anchor boxes. However, the authors in [7] have shown that anchor boxes are not sufficient to obtain accurate detection, and this could be caused by the down-sampling operation in the convolutions layer. A typical convolutional layer has a fixed scale and uses a fixed receptive field in the whole document. Thus, small tables risk being missed, which consequently increases the recall rate of object proposals; and (ii) at each location in the feature map, the RPN predicts the objectness score, which indicates whether the anchor is positive or negative: anchors with a high *IoU* overlap with the ground truth are classified as positives, otherwise they are considered as negatives. It was reported in [12] that filtering the majority of positive anchors alleviates the foreground–background class imbalance and drives the R-CNN to outperform other frameworks such as SSD [13] and YOLO [14]. However, the confusion problem remains with Faster R-CNN and stems from an important rate of false positives. This is simply because the objectness score do not precisely reflect the correct location in the region proposal. In other words, an anchor box may contain an object different from the interest object, but the later is classified as a positive anchor even though it is a negative anchor and its localization is not an object of interest. Consequently, this kind of anchor could degenerate the RPN with false positives and lead to the confusion problem. (iii) The RoI pooling layer suffers from the lack of accuracy caused during mapping region proposal coordinates on the feature map and using max pooling to aggregate features.

In order to remedy the underlined problems, we use a newly introduced detection method based on Faster-RCNN, called "DCTable", to detect and localize tables. The key contributions of this paper are the following:

- We use a dilated VGG-16 network for the feature extraction where we remove the downsampling (in max-pooling and strided convolution). This leads to the expansion of the receptive fields of the conv_4 and conv_5, thus obtaining more discriminative features and preventing both confused and missed detections.
- We leverage the great potential of weighted *IoU* in the correlated *IoU* balanced-loss functions [15] to improve the localization accuracy of the RPN and alleviate the confusion problem.
- We introduce the bilinear interpolation in the Faster R-CNN in order to ensure a mapping based on exact spatial locations and correctly align the extracted features with the input by replacing the typical RoI pooling with the RoIAlign layer.
- We evaluate the enhanced approach on four datasets using not only a Precision-Recall space, but also the ROC space to show how much our approach improves localization.

The remainder of this paper is organized as follows. Section 2 presents related works. Section 3 describes the details of our proposed methodology for table detection in scanned documents. Materials in terms of the used datasets and metrics are defined, respectively, in Section 4 and Section 5. The obtained results are discussed in Section 6. Finally, Section 7 concludes the paper.

## 2. Related Works

Research on table detection started in the 2000s, before the emergence of deep learning-based methods. This task was performed in a hand-crafted way using rules and heuristics [16,17]. Later, many machine learning techniques were used for table detection tasks, which led to a significant improvement of the table detection accuracy, as in [18,19,20]. When reviewing table detection related papers, we found that since 2017, a considerable amount of research effort was made using the groundbreaking object detector framework Faster R-CNN [7]. While some researchers proposed two- or multistage table detection processes where they were used to prepossess document images, others explored Faster R-CNN [7] with different backbones to perform table detection tasks.

### 2.1. Heuristics-Based Table Detection

Kienninger et al. [16] proposed the known T-recs system, which relies on word grouping into columns to identify table cells. These methods are outperformed by machine learning techniques. The authors in [17] introduced the first learning-based approach where they represented a document by a MXY tree from which they identified blocks with horizontal and vertical lines. By 2015, a new wave of introduced works defined table detection tasks in the form of object-detection problems and proved that this paradigm works efficiently for such tasks. In this context, table regions were located and extracted using local thresholds for word space and line height from scanned document images in [20]. An alternative approach was proposed in [21] and presented a regions of interest-based method and the spatial arrangement of extracted text blocks.

### 2.2. Learning-Based Table Detection

A table detection task was performed in [18] with the Hidden-Markov-Models and in [19] where the SVMs were applied to hand-crafted features. In 2017, the most first work [8] used Faster R-CNN [7] to pre-process data with the Euclidean distance transform, the linear distance transform and the max distance transform. Then, Faster R-CNN was fine-tuned to detect tabular regions. Another method based on pre-processing is proposed in [22], where authors assume that colors would boost the ability of Faster R-CNN in distinguishing table regions. For such, they use to feed a colored document image to Faster R-CNN. The proposed method applied a distance transform to the blue channel only and reached a good result with the fine-tuned Faster R-CNN based on a ResNet backbone [23]. NLPR-PAL, owner of the best method on ICDAR 2017 table detection tasks [24], is a multistage approach where authors start by classifying the connected component into text, figures and tables with SVM. Then, they merge the obtained figures and tables and apply Faster R-CNN to distinguish the connected component of tables from those of figures. Another research work [25] uses Faster R-CNN and combines it with the table corner locating method to remedy the problem of missed table boundaries. Furthermore, the authors in [10] adopted YOLOv3 [26] by including an anchor optimization strategy and two post processing methods to solve the problem of inaccurate edges detection, which directly affects the system performance.

We also notice that there are some works [27,28] that simply fine-tune Faster R-CNN to the table detection task. In the same context, Casado-Garcìa et al. [29] conduct a comprehensive study on the benefits of close domain fine tuning by comparing Mask R-CNN [9], Retina [12], SSD [13] and YOLO [14]. They show that in addition to solving the problem of data scarcity and avoiding overfitting, fine tuning from a close domain considerably improves the accuracy of the produced model.

Instead of typical convolutions, the deformable convolutions are used in many works in order to leverage the power of adapting the receptive field of the network to the size of the input table. The authors in [30] equip Faster R-CNN with a deformable ResNet-101 backbone. The deformable receptive field is also introduced to the RoI pooling in order to adapt its receptive fields to random scales and transformations of the input. The authors in [31] presented the first multistage deep neural network for table detection where the main structure of this network is based on the Cascade Mask R-CNN [32] with a composite backbone [33] having a deformable convolution for detecting tables in different scales. A novel backbone, the HybridTabNet (HTC) [34], was recently used in [35] for table detection task. The authors take advantage from this deformable backbone as a unified network for joint object detection and segmentation. In addition, CasTabDetectoRS [36] is another a novel table detection method that is based on Cascade Mask R-CNN [32] combined with Recursive Feature Pyramid Network [37] and Switchable Atrous Convolution [38] as backbones. An alternative approach to convolutional networks is proposed in [11]. The authors use Graph Neural Networks (GNN) for table table detection in invoices.

Most of the mentioned works in the field of table detection achieved significant results on a variety of datasets. However, and to the best of our knowledge, there are two important aspects of the table as an object that need to be studied more, which are the figure-like layout and the small size of the table.

## 3. Method

**Figure 1:**
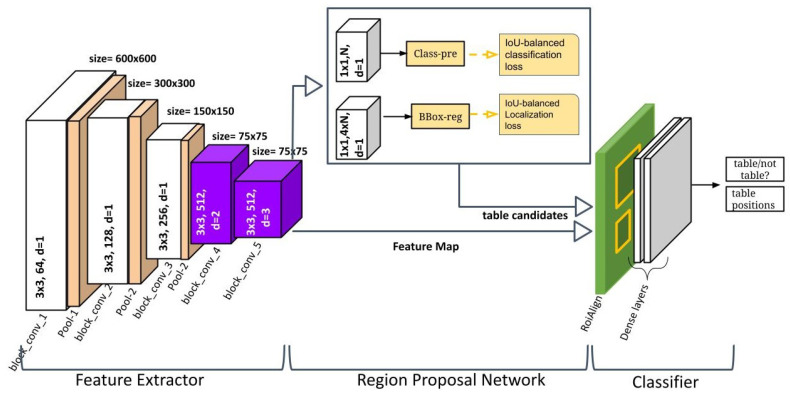
The DCTable architecture. In the first stage, the feature extractor is composed of strided convolutional layers in the three first blocks and where each one is followed by a pooling layer. The two other blocks are equipped with dilated convolutions where the used dilation rates are d=2 and d=3, respectively. Then, the RPN is trained with *IoU*-balanced loss. The final stage is composed of ROIALign layer and dense layers.

In this section, we illustrate the main contribution of this paper, which presents our proposed method, DCTable (as shown in Figure 1). An input document image is fed to the VGG-16 with dilated convolution layers in order to extract features. On top of these feature extractors, an RPN (Region Proposal Network) is constructed to simultaneously predict table region coordinates and objectness scores. This RPN is trained using high correlated *IoU*-balanced losses. Then, the obtained candidates are fed to the RoIAlign layer, which performs the bilinear interpolation on the mapping table region coordinates on the feature maps and pooling features.

### 3.1. Feature Extractor with Dilated Convolutions

Since the first implementation of Faster R-CNN [7], where the authors used VGG-16 [39] as the most deepest CNN, it becomes the default baseline backbone architecture. Moreover, the authors in [40] are the only ones who used dilated convolutions to build a VGG-16 [39] for tables and charts classification. Motivated by their results, we implement DCTable based on dilated VGG-16 along with the replacement of conventional convolutions with dilated ones. A dilated convolution is defined in [41] as a *d*-dilation convolution where *d* is the dilation factor:

(1) $$\left(F{*}_{d}k\right)=\sum_{s+dt=p}F\left(s\right)k\left(t\right)$$

where *k*: $R_{r}\to R$ is a discrete filter of size ${\left(2d+1\right)}^{2}$. If d=1, then the convolution is a 1−dilated convolution and it refers to the typical convolution. A convolution with a dilation factor d=1 exponentially expands the receptive field and drops the down-sampling operation to avoid loss of resolution.

**Figure 2:**
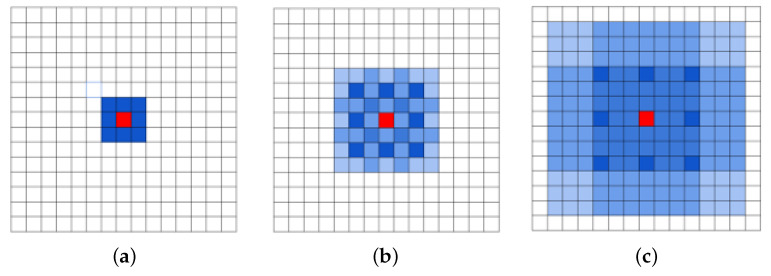
Impact of stacking dilated convolutions with different dilation rates on a 3 × 3 filter [42]: (**a**) $F_{1}$ is produced from $F_{0}$ by a 1-dilated convolution, (**b**) $F_{2}$ is produced from $F_{1}$ by a 2-dilated convolution, and (**c**) $F_{3}$ is produced from $F_{2}$ by a 3-dilated convolution.

As illustrated by Figure 1, the backbone is composed of strided convolutional layers in the three first blocks with d=1 and where each one is followed by a pooling layer. Thus, the size of the feature map decreases from 600×600 to 75×75. Then, we replace the typical convolutional layers in the con_4 and conv_5 with dilated ones where the used dilation rates are d=2 and d=3, respectively. We remove the pooling layers so the size of the feature map remains unchanged. It was shown in [42] that stacking dilated convolution kernels with a fixed dilation rate causes the gridding issue, which refers to losing important features in the feature map. In a dilated convolution, the receptive field covers only locations with non-zero padding. This problem was alleviated in [43], by removing the max-pooling in the model to reduce the high-amplitude and high-frequency. However, the problem is exacerbated on the top-most layers. That is why the HDC [42] came up to further reduce the gridding by using arbitrary dilation rates without using a common factor through the network as in [40], which could generate a sparse sample from the input and lead to missing relevant information. This is important not only for small tables, but also for big ones without adding extra blocks as in [43]. For this reason, we used three different dilation rates the backbone. In Figure 2, we represent transformations produced on a filter by applying dilated convolutions with increased dilation rates on this filter.

### 3.2. IoU-Balanced Loss for Optimizing Anchors

A RPN is a fully convolutional network that simultaneously predicts object bounds and objectness scores at each position [7]. The famous cross-entropy loss and smooth L1 are adopted, respectively, for its two branches and are defined in Equation (2).

(2) $$\begin{array}{c} L\left(p_{i},t_{i}\right)=\frac{1}{N_{cls}}\sum_{i}^{}L_{cls}\left(p_{i},\tilde{p_{i}}\right)+\lambda \frac{1}{N_{reg}}\sum_{i}^{}\tilde{p_{i}}L_{reg}\left(t_{i},\tilde{t_{i}}\right) \end{array}$$

The classification loss $L_{cls}$ is a logarithmic function over two classes, object and not object. The index of an anchor in a mini-batch is represented with *i*, and pi is the predicted probability of anchor *i* being an object. In the case where the anchor is positive, $\tilde{p_{i}}$ as the ground-truth label will be equal to 1, otherwise it will be 0.

For the regression loss, it is based on the robust loss function (smooth L1) where $t_{i}$ is a vector representing the four parameterized coordinates of the predicted bounding box, and $\tilde{t_{i}}$ is the vector of the ground-truth box associated with a positive anchor. However, the classification loss drives all the positive anchors to learn their high classification scores without considering their location quality. The regression loss $L_{reg}$ is also activated only for positive anchors [7]. Thus, this weak correlation between classification and regression loss functions affects localization accuracy and increases the number of false positives. To strengthen this correlation and enhance the localization accuracy in a one-stage object detection framework, *IoU*-balanced loss functions [15] use weighted positives examples based on their localization accuracy. The *IoU*-classification loss is defined as follows:

(3) $$\begin{array}{c} Loss_{cls}=\sum_{i\in Pos}^{N}\omega_{i}\left(IoU_{i}\right)*\mathrm{CE}\left(p_{i},{\hat{p}}_{i}\right)+\sum_{i\in Neg}^{M}\mathrm{CE}\left(p_{i},{\hat{p}}_{i}\right) \end{array}$$

This function (Equation (3)) is used to up-weight examples with high *IoU* and down-weight examples with low *IoU* as follows: Pos and Neg represent the sets of positive training examples and negative training examples, respectively. $p_{i}$ and ${\hat{p}}_{i}$ represent the predicted classification score and the corresponding ground truth label, respectively, with CE the cross-entropy loss. $IoU_{i}$ represents the regressed *IoU* for each regressed positive samples. $w_{i}\left(IoU_{i}\right)$ represents the assigned weights to positive samples and is defined in Equation (4).

(4) $$\omega_{i}\left(IoU_{i}\right)=IoU_{i}^{\eta }\frac{\sum_{i=1}^{N}\mathrm{CE}\left(p_{i},{\hat{p}}_{i}\right)}{\sum_{i=1}^{N}IoU_{i}^{n}\mathrm{CE}\left(p_{i},{\hat{p}}_{i}\right)}$$

In Equation (2), the loss function is driven by a positive sample because the weight of all the samples is restricted to be binary 1,0. Therefore, all the negative samples are suppressed since their weights are equal to 0. However, the *IoU*-loss classification function uses two properties of input anchors: the weight and the *IoU*, where the weight is assigned based on the *IoU*. Thus, different weights are assigned to all the input samples. In the mentioned equation, η controls to what extent the *IoU*-balanced classification loss focuses on examples with high *IoU* and suppresses examples with low *IoU*. For implementation, we fix η at 1.5 since, in the paper of [15], the detector achieves the best performance.

It is true that the Smooth L1 loss was used in [6] as robust against outliers compared to the Smooth L2 loss used in R-CNN [5]. According to the results of [44], the localization loss is driven by samples with low *IoU*, which represent outliers and dominate the gradients. Hence, there would be a significant degradation of the RPN performance. Motivated by this fact, *IoU*-balanced localization loss put more focus on inliers by assigning great weights to examples with high *IoU* and reducing weights of examples with low *IoU* as defined in Equation (5).

(5) $$Loss_{reg}=\sum_{i\in Pos}^{N}\sum_{m\in x,y,w,h}\omega_{i}\left(IoU_{i}\right)*smooth_{L1}\left(l_{i}^{m}-g_{i}^{m}\right)$$

where,

(6) $$\begin{array}{c} \omega_{i}\left(IoU_{i}\right)=\omega_{loc}*IoU_{i}^{\lambda } \end{array}$$

In the defined equation, while $\left(l_{i}^{x},l_{i}^{y},l_{i}^{w},l_{i}^{h}\right)$ represents the parameterized coordinates of the predicted box, $\left(g_{i}^{x},g_{i}^{y},g_{i}^{w},g_{i}^{h}\right)$ represents the parameterized coordinates of the corresponding ground truth box. λ is defined to control to what extent *IoU*-balanced regression focuses on inliers and suppresses outliers. As mentioned in [15], the best performance is obtained when λ=1.5, so we use this value in all our experiments.

### 3.3. RoIAlign in DCTable

**Figure 3:**
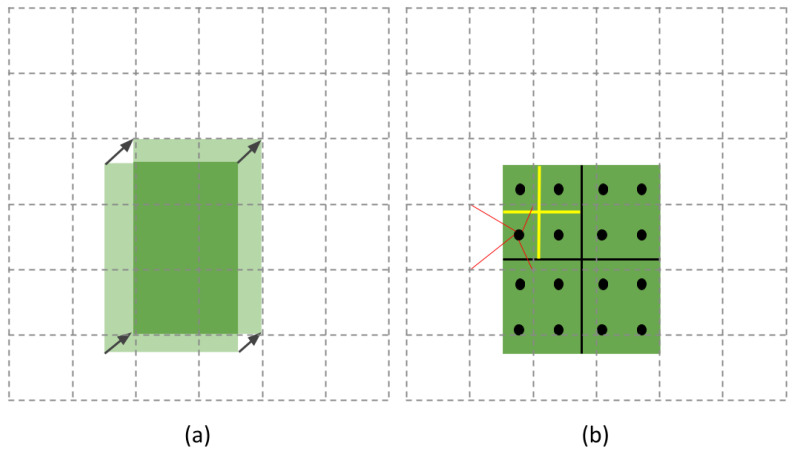
From Fast R-CNN to Mask R-CNN: (**a**) RoI-pooling layer and (**b**) RoIAlign layer.

Faster R-CNN was designed to perform an object detection task and return the positions of the predefined classes. The output of the first stage is a set of region candidates described by a bounding box (r, c, h, w) into a feature map, where (r, c) represent its top-left corner and (h, w) represent the height and width, respectively. In the second stage, the predicted coordinates are used by the RoI pooling layer. This layer was defined in [6] as a downsampling operation that pools over local features extracted from different image feature maps and generates small features of the size (H×W). The input of a RoI layer is, indeed, a set of feature maps containing object proposals where each one is described by the predicted coordinates, from the first stage, forming a bounding box (bbox). Let this bounding box be a tuple of float coordinates (x, y, h, w) where (x, y) represents its top-left corner and (h, w) the height and the width, respectively. In order to identify regions covered by RoI features meant to be pooled, the aforementioned coordinates are quantized into the discrete granularity of the feature map as shown in Figure 3. In other words, the RoI pooling rounds up every float coordinate to map the region proposal to the feature map and obtains a RoI with a size of h×w. The quantization is also performed on this RoI by dividing it into a k×k grid where k=h/H and k=w/W and the features of each subgrid are aggregated by a max pooling operation [9]. Figure 3a shows the evident misalignment caused by not only the quantizing-based mapping of the RoI to the feature map, but also dividing the RoIs into bins, so that the new position of the spatial coordinates impacts the bounding box accuracy.

**Figure 4:**
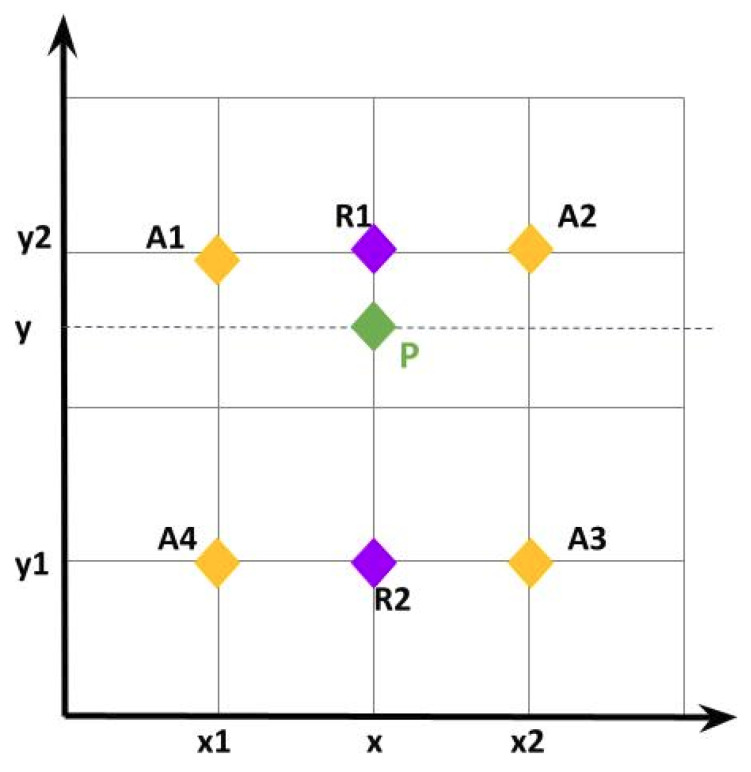
The bilinear interpolation.

Faster R-CNN was extended by the Mask R-CNN [9] with a branch to perform a pixel-level object instance segmentation by predicting an object mask in parallel with the segmentation masks on each RoI (Region of Interest) for bounding box recognition. To avoid the coarse misalignment produced by the RoI pooling, the authors of [9] also introduced the RoIAlign as a quantization-free layer that uses bilinear interpolation [45] to ensure a mapping based on exact spatial locations and correctly aligns the extracted features with the input. The RoIAlign cancels every quantization performed on any coordinates and the bins of the RoI. In Figure 3b, we represent a feature map by a dashed grid while the mapped RoI is represented by a green rectangle with solid lines. This RoI is divided into 2×2 bins where each bin contains four sampling points represented by dark dots. Using the bilinear interpolation, the value of each sampling point is computed using the bilinear interpolation from the nearby grid points on the feature map. Figure 4 shows that bilinear interpolation is performed through linear interpolation in two directions. The values of points *A*1, *A*2, *A*3, and *A*4 are known, and let P be the unknown point that will be computed as follows. Firstly, *R*1 is obtained by a linear interpolation of *A*1 and *A*2 applied in the *x* direction, also *R*2 is obtained by interpolating *A*4 and *A*3:

(7) $$f\left(R1\right)≃\frac{x_{2}-x}{x_{2}-x_{1}}\left(A1\right)+\frac{x-x_{1}}{x_{2}-x_{1}}f\left(A2\right)$$

(8) $$f\left(R2\right)≃\frac{x_{2}-x}{x_{2}-x_{1}}\left(A4\right)+\frac{x-x_{1}}{x_{2}-x_{1}}f\left(A3\right)$$

Then, *P* is obtained by a linear interpolation of *R*1 and *R*2 in the y direction:

(9) $$f\left(P\right)≃\frac{y_{2}-y}{y_{2}-y_{1}}f\left(R1\right)+\frac{y-y_{1}}{y_{2}-y_{1}}f\left(R2\right)$$

## 4. Datasets

In order to show the effectiveness of the proposed methodology, we evaluate our new model on publicly available datasets: ICDAR-POD2017, ICDAR-2019, Marmot, and RVL-CDIP.

### 4.1. ICDAR-POD2017

This dataset has been released for a competition (ICDAR-2017 POD) [24] focusing on specific page objects comprising the detection of tables from images. According to the competition paper, the dataset exhibits a good variety in object styles including formulae, tables, graphics and figures. There are 817 images containing 317 tables. In this paper, we used about 900 images where the table region is used as a positive example while the background (paragraphs, figures and equations) is considered a negative example.

### 4.2. ICDAR-2019

The cTDaR competition aims at investigating and comparing general methods that can reliably and robustly identify the table regions within a document image on the one hand, and the table structure on the other hand [46]. In the paper of the cTDAR competition [46], two datasets were introduced. While the first one was presented for table detection (TRACK A), the second one was for table recognition (TRACK B). Those datasets consist of modern printed documents and archival documents. In this paper, we use TRACK A to train and test our proposed approach.

### 4.3. Marmot

The Marmot Dataset contains 2000 pages in PDF format, where most of the examples are from research papers, and contains 958 table labels [47]. The dataset is composed of Chinese and English pages. The Chinese pages were selected from over 120 e-Books with diverse subject areas provided by Founder Apabi library, and no more than 15 pages were selected from each book, while the English pages were crawled from Citeseer website. The e-Book pages are mostly in a one-column layout, while the English pages are mixed with both one-column and two-column layouts. When reviewing table detection related papers, we found that all existing works, such as [30,35], trained their frameworks using ICDAR 2017-POD and took Marmot as a testing dataset for evaluation. Therefore, we follow in our experiments the same protocol and we used the cleaned version of this set published by [27] to evaluate our model fine-tuned on ICDAR 2017-POD.

### 4.4. RVL-CDIP

RVL-CDIP [48] contains 400,000 grayscale images, which are categorized into 16 classes with 25,000 images per class. We annotate the region tables and backgrounds (logo, text, etc) of only 600 invoices. We used the prepared set to train and evaluate the performance of our models on scanned documents with noise. So, we randomly split the prepared set into a training and test set. While 80% are used to train the model, the remaining 20% are used to evaluate the performance of our model on noisy data such as RVL CDIP.

## 5. Evaluation Metrics

Many performance metrics have been mentioned in the literature and have been used by researchers in the evaluation of table detection algorithms.

### 5.1. Precision-Recall Space

As with any learning method, the efficiency of any model is determined using measures such as true positive (*TP*), false positive (*FP*), true negative (*TR*) and false negative (*FN*). It is worth mentioning that the performance evaluations are always based on a trade-off between the true positive and true negative rate, and between recall and precision. Consequently, the *F*1-score is the harmonic mean of both recall and precision and is widely used in this domain.

(10) $$Recall=\frac{TP}{TP+FN}$$

(11) $$Precision=\frac{TP}{TP+FP}$$

(12) $$F1-score=2*\frac{Precision*Recall}{Precision+Recall}$$

In our experiments, we assess our models using the intersection over union (*IoU*) of the predicted bounding box against the ground truth ones, which is defined as:

(13) $$IoU=\frac{P\cap T}{P\cup T}$$

where *P* and *T* are the predicted bounding boxes and the ground truth regions, respectively.

### 5.2. ROC Space

We use Receiver Operator Characteristic (ROC) [49] curves that show how the number of correctly classified positive examples varies with the number of incorrectly classified negative examples. In ROC space, the False Positive Rate (FPR) and the True Positive Rate (TPR) are plotted on the x-axis and the y-axis, respectively. While the *FPR* indicates negative examples that are miss-classified as positive, the *TPR* measures the positive examples that are correctly classified.

(14) $$TPR=\frac{TP}{TP+FN}$$

(15) $$FPR=\frac{FP}{FP+TN}$$

## 6. Results and Discussion

This section provides details on the different experiments performed to train and evaluate our approach, DCTable.

In the experiment, we evaluate the effectiveness of our approach, DCTable, including the dilated convolutions, the *IoU* balanced loss and the RoIAlign. We implement four different models as follows:

- DCTable-A: a Faster R-CNN based on a simple VGG-16. We use the default implementation as in [6]. The output regions proposals are fed into the RoI Pooling layer. The RPN is trained using the typical loss function as defined in the original paper [7].
- DCTable-B: a Faster R-CNN based on a dilated VGG-16. We replaced conventional convolutions of the conv_4 and conv_5 with dilated ones where the used dilation rates are d=2 and d=3, respectively. The output region proposals are fed into the RoIALign layer. The RPN is trained using the typical loss function as defined in the original paper [7].
- DCTable-C: we replaced the typical loss function in the RPN in DCTable-A with the *IoU*-balanced loss function.
- DCTable: we replace the loss functions of the RPN in DCTable-B with the *IoU*-balanced loss function.

We used the pretrained weights of VGG-16 from ImageNet as the backbone of the Faster R-CNN. The training images are resized to 600 × 600 and we use for all models three different anchor ratios: 0.5, 1 and 2, and three different anchor scales: 128 × 128, 256 × 256 and 512 × 512. Our models are optimized for 2500 epochs (with 32 as batch size) using Adam as an optimizer starting from a learning rate as 0.00001. In order to avoid overfitting, we use random horizontal flips for data augmentation.

In all our experiments, training and testing were performed with Tensorflow on the Google Colab Pro platform, using a Tesla T4 GPU.

### Effectiveness of IoU-Balanced Loss

**Figure 5:**
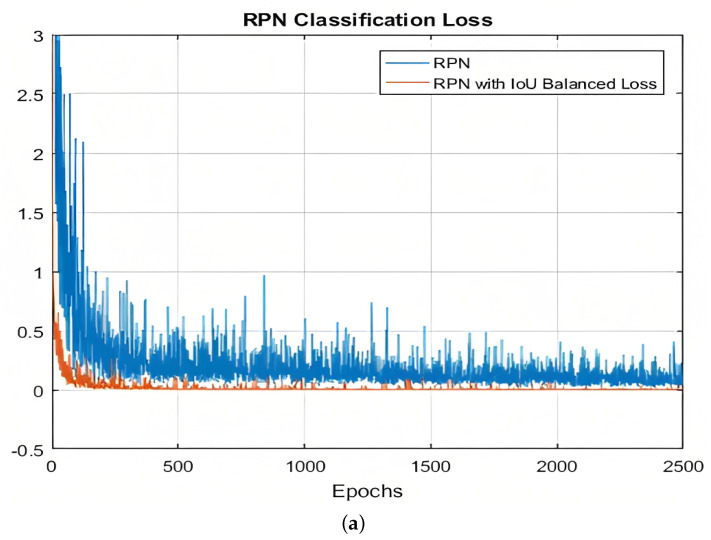

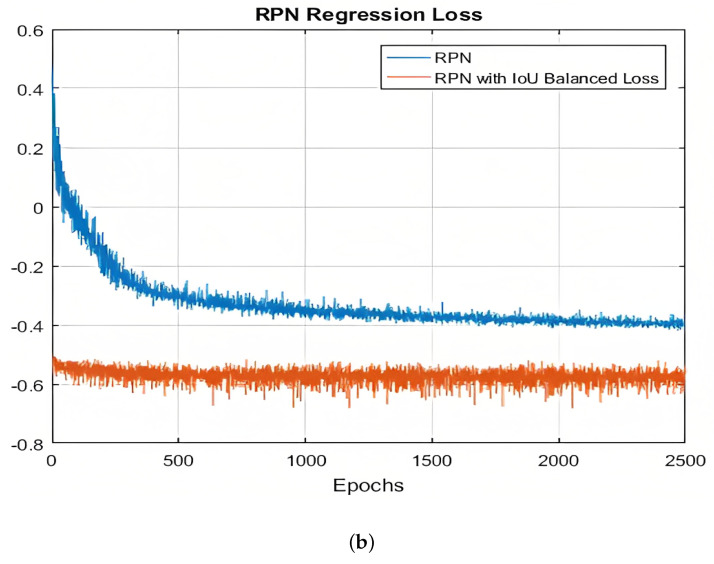
Loss function curves: (**a**) loss function classification: the typical loss function and the *IoU*-balanced loss, (**b**) loss function localization: the typical loss function and the *IoU*-balanced loss for localization.

As shown above, we set-up the loss functions of the RPN. So, we compare two different trained RPNs: RPN with the typical cross-entropy, which is regarded as the classification loss function, and the *IoU*-balanced classification loss, which is regarded as the regression loss. The changes of the different loss functions on classification and regression are shown, respectively, in Figure 5a,b during the training of the RPN. As the training progresses, the value of the loss function continuously decreases. The loss function stabilizes and reaches a minimum after the first 20 epochs. For the regression function, it is obvious in Figure 5b that it reaches a minimum quickly. Thus, it can be seen that *IoU*-balanced losses-based RPN has a higher convergence speed than the typical RPN in terms of all the performance indexes.

### 6.1. Test Performance on ICDAR2017

**Figure 6:**
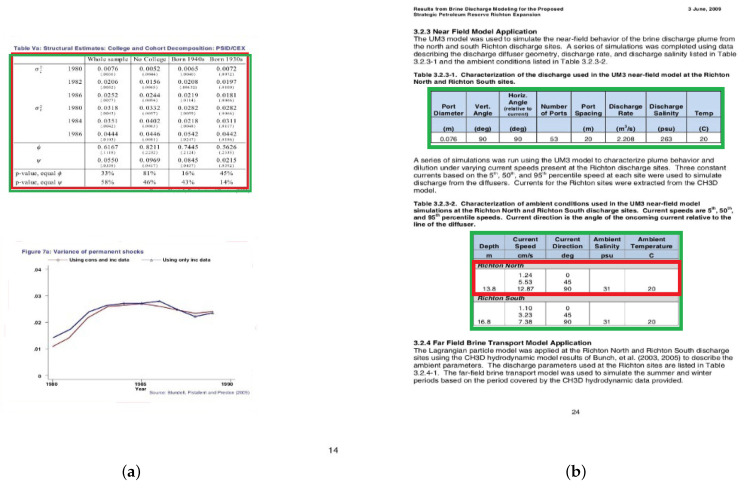
Some sample images from ICDAR 2017-POD showing: (**a**) True Positive, (**b**) false positive. The green region represents the ground truth bounding boxes while red region represents bounding boxes of detected regions.

**Figure 7:**
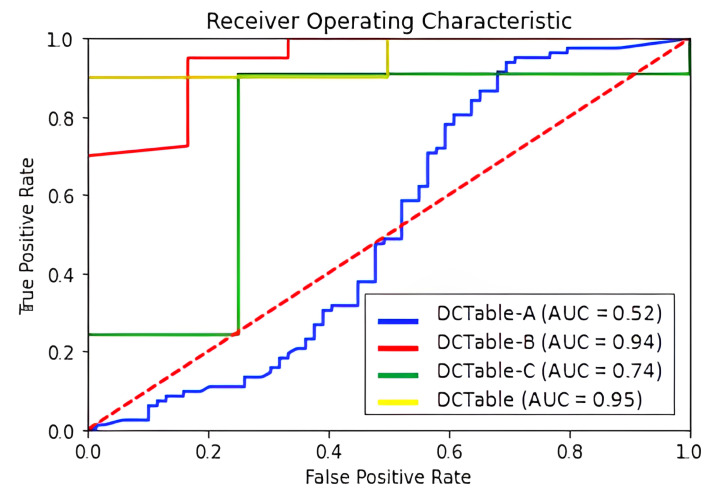
ROC curve for ICDAR 2017-POD dataset.

Table 1 reports the obtained results on ICDAR 2017 where we use two different *IoU* thresholds of 0.6 and 0.8. Thanks to the potential of the dilated convolutions and the RoIAlign, our method produced a tightly bounding box. It is obvious in the mentioned table that the DCTable-B is more accurate and enhances the *F*1-score by 4% at 0.8 *IoU* compared to DCTable-A. By introducing the *IoU*-balanced loss functions, and compared to DCTable-B, DCTable improved the *F*1-score by 0.8% at 0.8 *IoU*. The detected table in Figure 6 is the only under segmented region in the whole test set. At the same time, in ROC curve Figure 7, the DCTable achieves the best TPR since the AUC is of 95%. The AUC of DCTable-A is of 52%, which is caused by the confusion problem while, the AUC of DCTable-C is about 74%. This leads us to conclude the effectiveness of *IoU*-balanced loss in decreasing the FPR rate. At 0.6 *IoU*, our proposed DCTable reaches the best performance (an *F*1-score of 97.5%) compared to DeCNT and FastDetectors, which achieved an *F*1-score of 96.8% and 92.1%, respectively. Even at 0.8, we improve the *F*1-score by 3% (compared to [25]) and detect all the table corners without any extra post-processing.

### 6.2. Test Performance on ICDAR 2019

**Figure 8:**
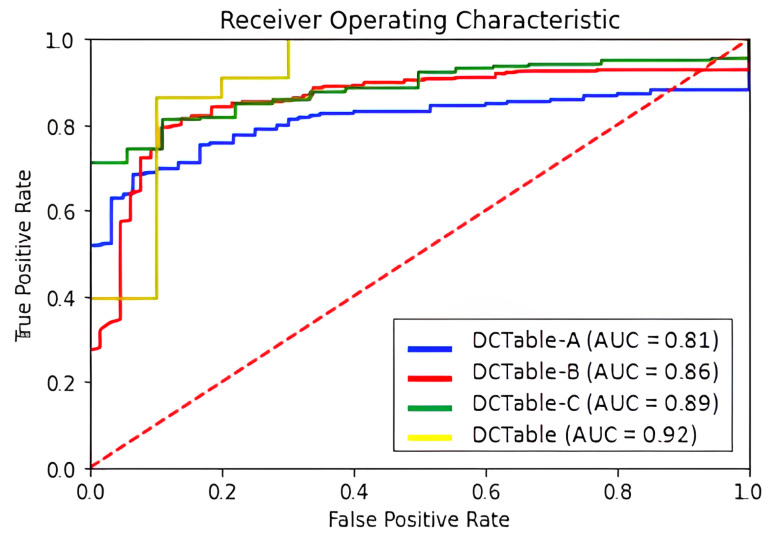
ROC curve for ICDAR 2019 dataset.

**Figure 9:**
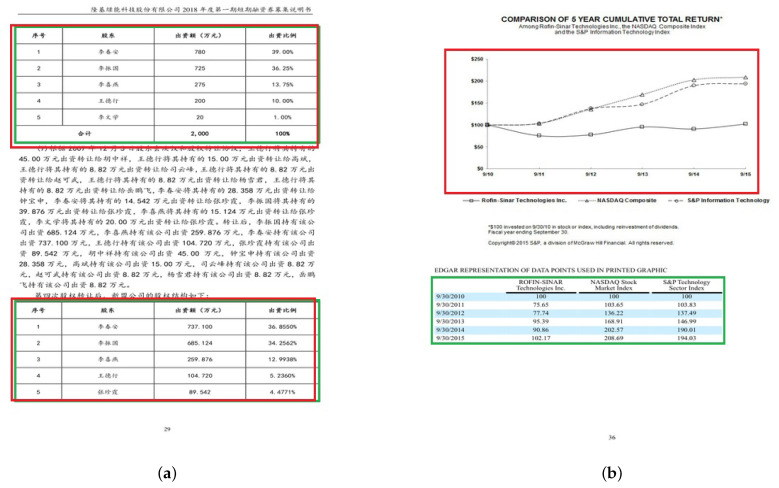
Some sample images from modern ICDAR 2019 showing: (**a**) true positive and (**b**) false positive. The green region represents the ground truth bounding boxes while the red region represents bounding boxes of detected regions.

The detection results on ICDAR 2019 are reported in Table 2. Without dilated convolutions, *IoU*-balanced loss and RoIAlign, the DCTable-A only achieves a 87.8% *F*1-score at 0.9 *IoU*. However, DCTable-B improved the *F*1-score by 2% since the recall has been increased. Basically, this improvement stems from the dilated convolutions and RoIAlign. Moreover, we found that the DCTable moves towards the disappearance of missed detections, but also represents the best achievable configuration of the Faster R-CNN in terms of recall-precision and the tightness of the boxes at 0.9 *IoU*. Even at 0.8 *IoU*, the DCTable achieves the best state of the art performances compared to NLPR-PAL [24] and Lenovo Ocean [46]. At 0.6 *IoU*, the DCTable has been able to successfully detect all the table regions and achieved a 98.5% *F*1-score. It is obvious in the ROC curve in Figure 8 that the DCTable has the best AUC, which is 92%. We observed that the recall decreases while increasing the *IoU*, thus it causes some missing detections. Compared to the recall, the low precision is caused by some under-segmented and over-segmented bounding boxes as represented in Figure 9b,c. The dilated convolutions with RoIAlign in DCTable-B improve the quality of detections from 81% to 86% in terms of accurate localization, but still suffer from the high FPR, which depends on the FP produced from the confusion problem.

### 6.3. Test Performance on Marmot

**Figure 10:**
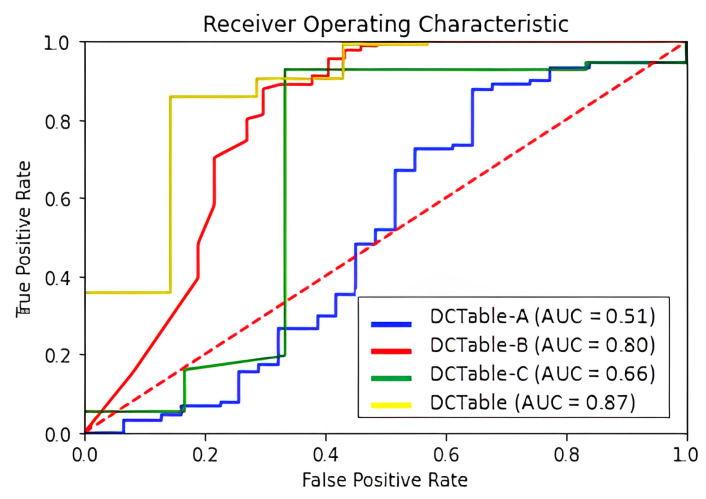
ROC curve for Marmot dataset.

**Figure 11:**
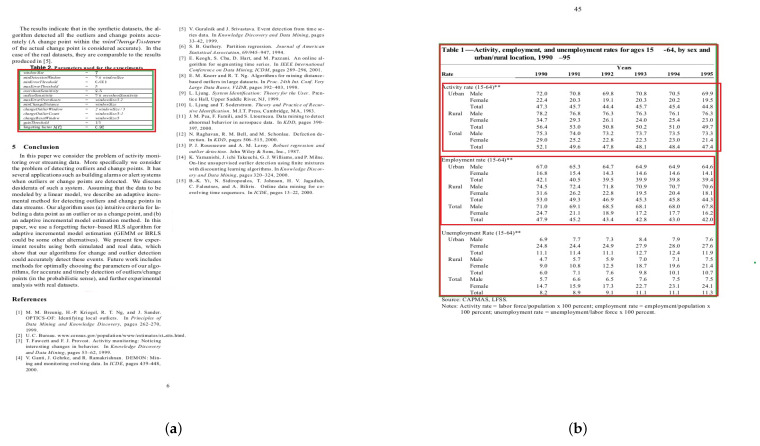
Some sample images from Marmot showing: (**a**) true positive and (**b**) false positive. The green region represents the ground truth bounding boxes while red region represents bounding boxes of detected regions.

The results in Table 3 show that DCTable-A fails to be accurate in table detection on the Marmot dataset. When comparing models DCTable-A and DCTable-C, we found that the precision has been increased by using *IoU*-balanced loss functions. So, this conduct proves that the weakness of localization and classification loss functions of the RPN is the harmful factor affecting the feature discrimination of DCTable-A. Consequently, and with the large variety in page layouts of the Marmot set, the model confuses between tabular regions and other objects such as figures and charts. The RoC curve also shows that DCTable-A suffers from high FPR compared to DCTable-C. Even DCTable-B was not able to handle the confusion problem (the precision is about 70.5%) while achieving an AUC of 80% and improved the recall, which is of 1 at 0.5 *IoU*. However, the DCTable came up with the dilated convolutions, high correlated *IoU*-balanced loss and RoIAlign to produce correct detections and achieve the best AUC in Figure 10, which is of 87%. Compared to the state-of-the-art, our DCTable achieves the best *F*1-score at both 0.5 and 0.9 *IoU* with, respectively, 96.6% and 96.9%. Figure 11 shows some of the errors that occurred during table detection such as false positive, but also correct detections.

### 6.4. Test Performance on RVL-CDIP

**Figure 12:**
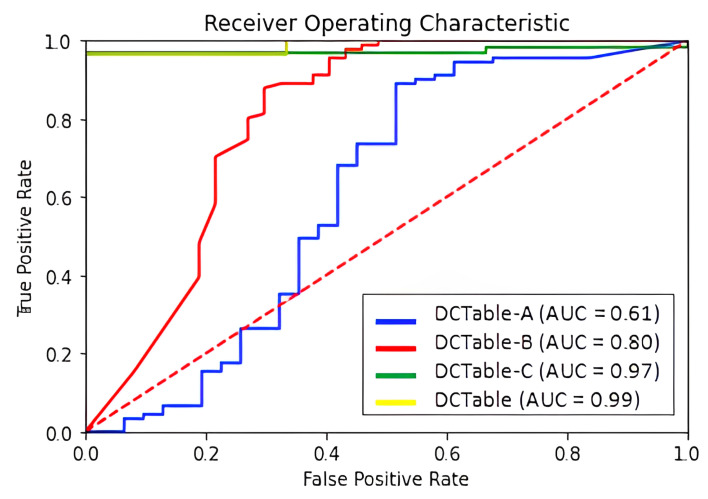
ROC curve for RVL-CDIP dataset.

**Figure 13:**
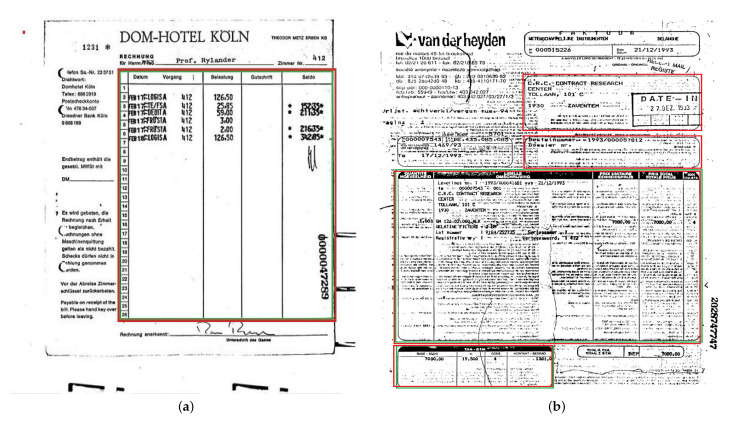
Some sample images from RVL-CDIP showing: (**a**) true positve and (**b**) false positive. The green region represents the ground truth bounding boxes while the red region represents bounding boxes of detected regions.

Despite the noise in the RVL-CDIP set, our models performed well on this dataset, as shown in Table 4. DCTable-B outperforms DCTable-A. This result is due to the increased number of overlapped detections. Furthermore, DCTable-A fails to detect tables in a document image with a high level of noise and achieve good results at 0.5 *IoU*. Additionally, it is obvious in Figure 12 that DCTable achieves the better TPR with an AUC of 99%. Figure 13 shows some of the errors that occurred during table detection.

### 6.5. Test Performance with Leave-One-Out Scheme of DCTable

In this section and inspired by [30,35], we present the cross-dataset performance of DCTable following a leave-one-out scheme. In order to evaluate the generalization capabilities of DCTable, we defined four schemes as follows:

- Scheme 1: DCTable is trained on a combining set composed of ICDAR 2019, Marmot and RVL CDIP and tested on ICDAR 2017.
- Scheme 2: DCTable is trained on a combining set composed of ICDAR 2017, Marmot and RVL CDIP and tested on ICDAR 2019.
- Scheme 3: DCTable is trained on a combining set composed of ICDAR 2017, ICDAR 2019 and RVL CDIP and tested on Marmot.
- Scheme 4: DCTable is trained on a combining set composed of ICDAR 2017, ICDAR 2019 and Marmot and tested on RVL CDIP.

We report the evaluation in Table 5, where we used the same *IoU* thresholds values 0.6, 0.8 and 0.9 to identify true positives. For scheme 1, the achieved *F*1-score decreased at 0.6 *IoU* by 1% compared to Table 1, but increased at 0.8 *IoU* by 1.2% to be the state-of the art result on ICDAR 2017. We found that the *F*1-score slightly decreased for scheme 2 and 3 compared to Table 2 and Table 3. For the forth scheme and compared to the results in Table 4, the *F*1-score dropped out because RVL CDIP is quite different from 97.3% to 75%, the combined training set of ICDAR 2017, ICDAR 2019 and Marmot in terms of the quality of scanned documents and also type (a set of noisy invoices). By analyzing the failure cases in all the test sets for the other schemes (1, 2 and 3), we found some detections drawn very far from the table border. In other cases, the empty regions of the tables are not inside the returned bounding boxes.

## 7. Conclusions and Future Work

In this paper, we propose a new detection method, called "DCTable", for table detection where we combine the dilated convolution layers with RoIAlign. The RPN is trained using the *IoU*-balanced loss functions in order to improve localization accuracy. Experiments on public datasets show that our model, DCTable, generalized well on a variety of documents. By increasing the dilation rate in the backbone, we found a significant improvement in the recall. Additionally, using the bilinear interpolation based RoIAlign, a suitable bounding box is obtained for most of the detected tables. Moreover, training the RPN using *IoU*-balanced loss contributes to enhancing the accuracy of the localization by decreasing the false positive rates.

In this paper, our work yields interesting results by improving the *F*1-score on ICDAR-2017, ICDAR 2019 and Marmot. However, it still suffers from some localization errors on those datasets that may be caused by the lack of some visual cues or missed global information during feature extraction. As future work, we will pursue the search with attention mechanism [50] in order to improve the CNN performance during training and predictions on large scale datasets.

## Figures and Tables

**Table 1:** Evaluation on ICDAR 2017-POD.

Models	*IoU*	P	R	*F*1-Score
DCTable-A	0.6	0.891	0.937	0.913
	0.8	0.946	0.909	0.927
DCTable-B	0.6	0.919	1	0.958
	0.8	0.937	1	0.967
DCTable-C	0.6	0.911	0.911	0.911
	0.8	0.953	0.911	0.932
DCTable	0.6	0.952	1	0.976
	0.8	0.975	0.975	0.975
HustVision [24]	0.6	0.071	0.959	0.132
FastDetectors [24]		0.903	0.940	0.921
NLPR-PA L [24]		0.968	0.953	0.960
DeCNT [30]		0.965	0.971	0.968
CDeC-Net [31]		0.977	0.931	0.954
HybridTabNet [35]		0.882	0.997	0.936
CasTabDetectoRS [36]		0.972	0.941	0.956
HustVision [24]	0.8	0.062	0.836	0.115
FastDetectors [24]		0.879	0.915	0.896
NLPR-PAL [24]		0.958	0.943	0.951
DeCNT [30]		0.946	0.952	0.949
CDeC-Net [31]		0.970	0.924	0.947
HybridTabNet [35]		0.887	0.994	0.933
CasTabDetectoRS [36]		0.962	0.932	0.947
(Sun et al., 2019) [25]	0.832	0.943	0.956	0.949

**Table 2:** Evaluation on ICDAR 2019.

Models	*IoU*	P	R	*F*1-Score
DCTable-A	0.6	0.834	0.899	0.865
	0.8	0.866	0.887	0.876
	0.9	0.890	0.866	0.878
DCTable-B	0.6	0.828	0.929	0.875
	0.8	0.855	0.929	0.890
	0.9	0.869	0.926	0.896
DCTable-C	0.6	0.866	0.869	0.868
	0.8	0.896	0.851	0.873
	0.9	0.908	0.827	0.866
DCTable	0.6	0.971	1	0.985
	0.8	0.983	0.996	0.989
	0.9	0.983	0.991	0.987
TableRadar [46]	0.8	0.950	0.940	0.945
NLPR-PAL [24]		0.930	0.930	0.930
Lenovo Ocean [46]		0.880	0.860	0.870
CDeC-Net [31]		0.953	0.934	0.944
HybridTabNet [35]		0.920	0.933	0.928
CasTabDetectoRS [36]		0.964	0.988	0.976
TableRadar [46]	0.9	0.900	0.890	0.895
NLPR-PAL [24]		0.860	0.860	0.860
Lenovo Ocean [46]		0.820	0.810	0.815
CDeC-Net [31]		0.922	0.904	0.913
HybridTabNet [35]		0.895	0.905	0.902
CasTabDetectoRS [36]		0.928	0.951	0.939

**Table 3:** Evaluation on Marmot.

Models	*IoU*	P	R	*F*1-Score
DCTable-A	0.5	0.708	0.966	0.817
	0.9	0.776	0.941	0.850
DCTable-B	0.5	0.705	1	0.827
	0.9	0.778	0.901	0.891
DCTable-C	0.5	0.898	0.946	0.922
	0.9	0.945	0.929	0.937
DCTable	0.5	0.933	1	0.966
	0.9	0.969	0.971	0.969
DeCNT [30]	0.5	0.946	0.849	0.895
CDeC-Net [31]		0.975	0.930	0.952
HybridTabNet [35]		0.962	0.961	0.956
CasTabDetectoRS [36]		0.952	0.965	0.958
CDeC-Net [31]	0.9	0.774	0.765	0.769
HybridTabNet [35]		0.900	0.903	0.901
CasTabDetectoRS [36]		0.906	0.901	0.904

**Table 4:** Evaluation on RVL-CDIP.

Models	*IoU*	P	R	*F*1-Score
DCTable-A	0.5	0.607	0.774	0.680
	0.8	0.635	0.734	0.681
DCTable-B	0.5	0.905	1	0.950
	0.8	0.948	1	0.974
DCTable-C	0.5	0.926	0.984	0.955
	0.8	0.955	0.984	0.969
DCTable	0.5	0.948	1	0.973
	0.8	0.964	1	0.982

**Table 5:** Evaluation with leave-one-out scheme of DCTable on ICDAR2017, ICDAR 2019, Marmot and RVL CDIP.

Scheme	Test Datasets	*IoU*	P	R	*F*1-Score
Scheme 1	ICDAR 2017	0.6	0.978	0.953	0.965
		0.8	0.981	0.995	0.987
Scheme 2	ICDAR 2019	0.6	0.961	0.959	0.959
		0.8	0.953	0.937	0.944
		0.9	0.921	0.950	0.935
Scheme 3	Marmot	0.5	0.854	0.884	0.868
		0.9	0.913	0.9	0.906
Scheme 4	RVL-CDIP	0.5	0.72	0.79	0.75
		0.8	0.68	0.73	0.70

## Data Availability

All data used in this work are available publicly. See Section 4 for the sources of the data used.

## References

[1] Marinai S. (2008). Introduction to Document Analysis and Recognition. Studies in Computational Intelligence.

[2] Faisal S., Smith R. Table detection in heterogeneous documents. Proceedings of the 9th IAPR International Workshop on Document Analysis Systems.

[3] Hashmi K.A., Liwicki M., Stricker D., Afzal M.A., Afzal M.A., Afzal M.Z. (2021). Current Status and Performance Analysis of Table Recognition in Document Images with Deep Neural Networks. IEEE Access.

[4] Bhowmik S., Sarkar R., Nasipuri M., Doermann D. (2018). Text and non-text separation in offline document images: A survey. Int. J. Doc. Anal. Recognit..

[5] Girshick R., Donahue J., Darrell T., Malik J. Rich feature hierarchies for accurate object detection and semantic segmentation. Proceedings of the 2014 IEEE Conference on Computer Vision and Pattern Recognition.

[6] Girshick R. Fast R-CNN. Proceedings of the 2015 IEEE International Conference on Computer Vision (ICCV).

[7] Ren S., He K., Girshick R., Sun J. (2017). Faster R-CNN: Towards real-time object detection with region proposal networks. IEEE Trans. Pattern Anal. Mach. Intell..

[8] Gilani A., Qasim S.R., Malik I., Shafait F. Table detection using deep learning. Proceedings of the 14th IAPR International Conference on Document Analysis and Recognition (ICDAR).

[9] He K., Gkioxari G., Dollár P., Girshick R. Mask R-CNN. Proceedings of the IEEE International Conference on Computer Vision (ICCV).

[10] Huang Y., Yan Q., Li Y., Chen Y., Wang X., Gao L., Tang Z. A yolo-based table detection method. In Proceeding of the 2019 International Conference on Document Analysis and Recognition (ICDAR).

[11] Riba P., Dutta A., Goldmann L., Fornés A., Ramos O., Lladós J. Table detection in invoice documents by graph neural networks. Proceedings of the 2019 International Conference on Document Analysis and Recognition (ICDAR).

[12] Lin T., Goyal P., Girshick R., He K., Dollár P. Focal loss for dense object detection. Proceedings of the 2017 IEEE International Conference on Computer Vision.

[13] Liu W., Anguelov D., Erhan D., Szegedy C., Reed S., Fu C., Berg A.C. SSD: Single shot multibox detector. Proceedings of the 2016 European Conference on Computer Vision.

[14] Redmon J., Divvala S., Girshick R., Farhadi A. You only look once: Unified, real-time object detection. Proceedings of the 2016 IEEE Conference on Computer Vision and Pattern Recognition (CVPR).

[15] Wu S., Yang J., Wang X., Li X. (2022). IoU-balanced loss functions for single-stage object detection. Pattern Recognit. Lett..

[16] Kieninger (1998). T. Table structure recognition based on robust block segmentation. Doc. Recognit..

[17] Cesarini F., Marinai S., Sarti L., Soda G. (2002). Trainable table location in document images. Proceedings of the 2002 International Conference on Pattern Recognition.

[18] e Silva A.C. Learning rich hidden markov models in document analysis: Table location. Proceedings of the 2009 International Conference on Document Analysis and Recognition.

[19] Kasar T., Barlas P., Adam S., Chatelain C., Paquet T. Learning to detect tables in scanned document images using line information. Proceedings of the 12th International Conference on Document Analysis and Recognition.

[20] Jahan M.A.C.A., Ragel R.G. Locating tables in scanned documents for reconstructing and republishing. Proceedings of the 7th International Conference on Information and Automation for Sustainability.

[21] Tran D.N., Tran T.A., Oh A., Kim S.H., Na I.S. (2015). Table detection from document image using vertical arrangement of text blocks. Int. J. Contents.

[22] Saman A., Faisal S. Table detection in document images using foreground and background features. Proceedings of the 2018 Digital Image Computing: Techniques and Applications (DICTA).

[23] He K., Zhang X., Ren S., Sun J. Deep residual learning for image recognition. Proceedings of the 2016 International Conference on Computer Vision and Pattern Recognition.

[24] Gao L., Yi X., Jiang Z., Hao L., Tang Z. ICDAR 2017 competition on page object detection. Proceedings of the 14th IAPR International Conference on Document Analysis and Recognition (ICDAR).

[25] Zhu N.S.Y., Hu X. (2019). Faster R-CNN based table detection combining corner locating. Proceedings of the 2019 International Conference on Document Analysis and Recognition (ICDAR).

[26] Redmon J., Farhadi A. (2018). Yolov3: An incremental improvement. arXiv.

[27] Schreiber S., Agne S., Wolf I., Dengel A., Ahmed S. (2017). DeepDeSRT: Deep learning for detection and structure recognition of tables in document images. Proceedings of the 14th IAPR International Conference on Document Analysis and Recognition (ICDAR).

[28] Li M., Cui L., Huang S., Wei F., Zhou M., Li Z. Tablebank: Table benchmark for image-based table detection and recognition. Proceedings of the 12th Language Resources and Evaluation Conference.

[29] Casado-García Á., Domínguez C., Heras J., Mata E., Pascual V. (2020). The benefits of close-domain fine-tuning for table detection in document images. International Workshop on Document Analysis Systems.

[30] Siddiqui S.A., Malik M.I., Agne S., Dengel A., Ahmed S. (2018). DeCNT: Deep deformable cnn for table detection. IEEE Access.

[31] Agarwal M., Mondal A., Jawahar C.V. CDeC-NET: Composite deformable cascade network for table detection in document images. Proceedings of the 2021 International Conference on Pattern Recognition (ICPR).

[32] Cai Z., Vasconcelos N. (2019). Cascade R-CNN: High quality object detection and instance segmentation. IEEE Trans. Pattern Anal. Mach. Intell..

[33] Liu Y., Wang Y., Wang S., Liang T., Zhao Q., Tang Z., Ling H. (2020). CBNet: A novel composite backbone network architecture for object detection. Proc. Int. Conf. Artif. Intell..

[34] Chen K., Pang J., Wang J., Xiong Y., Li X., Sun S., Feng W., Liu Z., Shi J., Ouyang W. Hybrid task cascade for instance segmentation. Proceedings of the IEEE/CVF Conference on Computer Vision and Pattern Recognition.

[35] Nazir D., Hashmi K.A., Pagani A., Liwicki M., Stricker D., Afzal M.Z. (2021). HybridTabNet: Towards Better Table Detection in Scanned Document Images. Appl. Sci..

[36] Hashmi K.A., Pagani A., Liwicki M., Stricker D., Afzal M.Z. (2021). CasTabDetectoRS: Cascade Network for Table Detection in Document Images with Recursive Feature Pyramid and Switchable Atrous Convolution. J. Imaging.

[37] Qiao S., Chen L.C., Yuille A. DetectoRS: Detecting objects with recursive feature pyramid and switchable atrous convolution. Proceedings of the 2021 IEEE/CVF Conference on Computer Vision and Pattern Recognition.

[38] Chen L.-C., Papandreou G., Kokkinos I., Murphy K., Yuille A.L. (2018). Deeplab: Semantic image segmentation with deep convolutional nets, atrous convolution, and fully connected crfs. IEEE Trans. Pattern Anal. Mach. Intell..

[39] Liu S., Deng W. Very deep convolutional neural network based image classification using small training sample size. Proceedings of the 3rd IAPR Asian Conference on Pattern Recognition (ACPR).

[40] Isaak K., Pino C., Palazzo S., Rundo F., Giordano D., Messina P., Spampinato C. A saliency-based convolutional neural network for table and chart detection in digitized documents. Proceedings of the 2019 International Conference on Image Analysis and Processing.

[41] Fisher Y., Koltun V. Multi-scale context aggregation by dilated convolutions. Proceedings of the 15th IEEE International Conference on Signal Processing (ICSP).

[42] Wang P., Chen P., Yuan Y., Liu D., Huang Z., Hou X., Cottrell G. Understanding convolution for semantic segmentation. Proceedings of the 2018 IEEE winter conference on applications of computer vision (WACV).

[43] Fisher Y., Vladlen K., Thomas F. Dilated residual networks. Proceedings of the 2017 IEEE Conference on Computer Vision and Pattern Recognition.

[44] Zhu L., Xie Z., Liu L., Tao B., Tao W. (2021). Iou-uniform R-CNN: Breaking through the limitations of RPN. Pattern Recognit..

[45] Max J., Simonyan K., Zisserman A. Spatial transformer networks. Proceedings of the 28th International Conference on Neural Information Processing Systems.

[46] Gao L., Huang Y., Déjean H., Meunier J., Yan Q., Fang Y., Kleber F., Lang E. ICDAR 2019 competition on table detection and recognition (cTDaR). Proceedings of the 2019 International Conference on Document Analysis and Recognition (ICDAR).

[47] Fang J., Tao X., Tang Z., Qiu R., Liu Y. Dataset, ground-truth and performance metrics for table detection evaluation. Proceedings of the 10th IAPR International Workshop on Document Analysis Systems.

[48] Chris T., Martinez T. Analysis of convolutional neural networks for document image classification. Proceedings of the 14th IAPR International Conference on Document Analysis and Recognition (ICDAR).

[49] Jesse D., Goadrich M. The relationship between Precision-Recall and ROC curves. Proceedings of the 23rd International Conference on Machine Learning.

[50] Vaswani A., Shazeer N., Parmar N., Uszkoreit J., Jones L., Gomez A.N., Kaiser Ł., Polosukhin I. Attention is all you need. Proceedings of the 31st International Conference on Neural Information Processing Systems.

